# Association Analysis Revealed That *TaPYL4* Genes Are Linked to Plant Growth Related Traits in Multiple Environment

**DOI:** 10.3389/fpls.2021.641087

**Published:** 2021-08-11

**Authors:** Yinghong Xue, Jingyi Wang, Xinguo Mao, Chaonan Li, Long Li, Xi Yang, Chenyang Hao, Xiaoping Chang, Runzhi Li, Ruilian Jing

**Affiliations:** ^1^College of Agronomy, Shanxi Agricultural University, Jinzhong, China; ^2^National Key Facility for Crop Gene Resources and Genetic Improvement, Institute of Crop Sciences, Chinese Academy of Agricultural Sciences, Beijing, China

**Keywords:** association analysis, functional molecular marker, ABA receptor, *TaPYL4*, plant growth related-traits, wheat

## Abstract

Abscisic acid (ABA), one of phytohormones, plays an important regulatory role in plant growth and development. ABA receptor *PYL4* (pyrabactin resistance 1-like 4) was previously detected to be involved in plant response to a variety of stresses. *TaPYL4* overexpression could enhance wheat (*Triticum aestivum*) drought resistance. In order to further investigate TaPYL4’s role in regulating development of other major agronomic traits in wheat, genes of *TaPYL4-2A*, *TaPYL4-2B*, and *TaPYL4-2D* were cloned from wheat, respectively. Polymorphism analysis on *TaPYL4* sequences revealed that encoding regions of the three genes were highly conserved, without any SNP (single nucleotide polymorphism) presence. However, nine SNPs and four SNPs were identified in the promoter regions of *TaPYL4-2A* and *TaPYL4-2B*, respectively. Functional molecular markers were developed based on these polymorphisms, which were then used to scan a natural population of 323 common wheat accessions for correlation analysis between genotype and the target phenotypic traits. Both *TaPYL4-2A* and *TaPYL4-2B* markers were significantly correlated with plant growth-related traits under multiple environments (well-watered, drought and heat stress treatments). The additive effects of *TaPYL4-2A* and *TaPYL4-2B* were verified by the combinational haplotype (*Hap*-AB1∼*Hap*-AB4) effects determined from field data. *Cis*-acting elements were analyzed in the promoters of *TaPYL4-2A* and *TaPYL4-2B*, showing that a TGA-element bound by ARFs (auxin response factors) existed only in *Hap*-2A-1 of *TaPYL4-2A*. Gene expression assays indicated that *TaPYL4-2A* was constitutively expressed in various tissues, with higher expression in *Hap*-2A-1 genotypes than in *Hap*-2A-2 materials. Notably, TaARF4 could act as *TaPYL4-2A* transcription activator in *Hap*-2A-1 materials, but not in *Hap*-2A-2 genotypes. Analysis of geographic distribution and temporal frequency of haplotypes indicated that *Hap*-AB1 was positively selected in wheat breeding in China. Therefore, *TaPYL4-2A* and *TaPYL4-2B* could be a valuable target gene in wheat genetic improvement to develop the ideal plant architecture.

## Introduction

Wheat (*Triticum aestivum*) is one of the most important food crops in the world, and also the main grain crop in China. The global demand of food is increasing greatly with social development and human population growth. Enhancement of wheat yield plays an important role in ensuring world food security. To increase wheat grain yield, gene mining and innovation of germplasm with superior agronomic traits are always the key research fields in wheat breeding. During the “Green Revolution” of 1960s, the discovery of semi-dwarf genes led to a dramatic increase in wheat yields (Peng et al., 1999; Saville et al., 2012). Despite many regulator genes of plant height were reported such as *TaCOLD1*, *Rht18*, and *Rht12* (Van De Velde et al., 2017; Dong et al., 2019; Buss et al., 2020; Tang et al., 2020), other important genes associated with wheat plant height remain to be identified. Combined with previous findings, exploiting the excellent genetic resources of wheat is the most economical and effective way to breed new varieties with higher grain yields.

Abscisic acid (ABA) is a key factor in balancing the metabolism of endogenous hormones and growth-related active substances in plants (Li et al., 2012). Early studies evidence that ABA can bind to ABA receptor, and such binding causes PP2Cs (clade A type 2C protein phosphatases) to lose phosphatase activity. The inhibition of PP2Cs enables activation of SnRK2s (sucrose non-fermenting 1-related protein kinase 2s) which consequently direct phosphorylation of downstream transcription factor AREB/ABFs (ABA-responsive element-binding factors) to induce the expression of ABA-responsive genes in the ABA signal transduction pathway (Fujii et al., 2009; Li et al., 2013; Vishwakarma et al., 2017; Zhao et al., 2020). Thus, ABA receptor plays a crucial role in the ABA signal transduction pathway (Park et al., 2009; Dittrich et al., 2019).

In *Arabidopsis*, the pyrabactin resistance 1 (PYR1)/PYR1-like (PYL)/regulatory components of the ABA receptor (RCAR) family of proteins contains 14 proteins (PYR1 and PYL1-13) with highly homologous sequences and structures (Nakashima et al., 2009; Zhao et al., 2018). The properties, structures and functions of PYLs have been extensively studied (Ma et al., 2009; Klingler et al., 2010; Hao et al., 2011b). There is increasing evidence that PYLs have a variety of functions in plant species such as *Arabidopsis*, rice and tomato. For instance, among the six PYL members expressed in the guard cells, PYL4 and PYL5 are involved in CO_2_ response (Dittrich et al., 2019). PYL9 could improve drought resistance and promote leaf senescence in *Arabidopsis* and rice (Zhao et al., 2016). PYL1, PYL4 and PYL6 in rice play a particularly important role in regulating plant growth (Miao et al., 2018). PYL5 in rice could increase drought tolerance and concurrently retard plant growth (Kim et al., 2014). Moreover, a recent report indicated that *TaPYL4* was highly acted in wheat tolerance to drought (Mega et al., 2019). However, the role of TaPYL4-mediated plant growth and development remains to be explored.

Candidate-gene association study (CGAS) is a kind of analysis based on the sequence of candidate genes. CGAS includes identification of the sequence polymorphism in the target gene and a correlation analysis between the polymorphism and the phenotypic traits, aiming to obtain the allelic variation affecting the target traits. Particularly, CGAS is used to analyze the correlation between different haplotypes and phenotypes to identify the excellent haplotype combinations responsible for the traits (Su et al., 2018). This tool is also used to excavate the excellent alleles with positive contribution to the target traits in germplasm resources. Functional markers can be directly used in detecting allele types of target genes, distinguishing different alleles and predicting phenotypic traits (Wang et al., 2019a). Moreover, functional markers can be used as the ideal molecular markers in marker-assisted breeding (Wang et al., 2020). With the development of functional genomics and genetic engineering, many functional molecular markers have been developed, providing abundant genetic markers for wheat molecular breeding.

In this study, gene sequencing was employed to examine polymorphism within *TaPYL4* locus (*TaPYL4-2A*, *TaPYL4-2B*, and *TaPYL4-2D*) in a populations consisting of 32 widely varied wheat germplasm. Functional markers were developed for *TaPYL4-2A* and *TaPYL4-2B* based on the *TaPYL4* polymorphism. These markers were then used to scan a natural population of 323 common wheat accessions for correlation analysis between genotypes and phenotypic traits. *TaPYL4-2A* and *TaPYL4-2B* were identified to be significantly correlated with plant growth-related traits. Furthermore, the additive effects of *TaPYL4-2A* and *TaPYL4-2B* were detected. Distribution of these haplotypes among the population consisting of wheat varieties from China was also examined, showing that *Hap*-AB1 was positive selected in wheat breeding. This work provides theoretical basis for wheat genetic improvement to obtain the ideal plant architecture, benefiting our understanding of ABA receptor upstream signaling network in plant morphogenesis.

## Materials and Methods

### Plant Materials

A common wheat (*Triticum aestivum*) variety, the Chinese Spring, was used for cloning of *TaPYL4* gene sequences. Thirty-two wheat accessions (Supplementary Table 1) with wide variation screened by 209 SSR markers (Zhang et al., 2013) were used to get gene sequences for detecting SNPs of *TaPYL4s.*

Three wheat populations were employed in this research. Population 1 (323 accessions) (Wang et al., 2019a) was used for agronomic character analysis. This population consisting of 275 modern cultivars, 36 advanced lines, and 12 landraces was also employed for association analysis. Population 2 and Population 3 consisted of 157 landraces and 348 modern cultivars (Zhang et al., 2002), respectively. The latter two populations were mainly from a Chinese wheat microcore germplasm, accounting for more than 70% of the genetic diversity of the whole Chinese wheat germplasm resources (Hao et al., 2011a). Haplotype frequencies among 10 wheat-producing areas in China were determined using Population 2 and Population 3 (Zhang et al., 2002).

### Management of Growth Conditions and Determination of Agronomic Traits

Population 1 was planted in 2015–2017 in Shunyi (40°23′N; 116°56′E) and Changping (40°13′N; 116°13′E) field experiment stations of the Institute of Crop Sciences, Chinese Academy of Agricultural Sciences, Beijing. Field water management was divided into two conditions: rain-fed (drought stressed, DS) and well-watered (WW). DS referred to that no artificial irrigation was given in the whole growing season of wheat, and the growth was completely fed by rain. WW was designed to irrigate the field with 750 m^3^ha^–1^ (75 mm) water at pre-wintering, jointing, flowering, and grain-filling stages, respectively. Moreover, a heat stress experiment (HS) was applied to wheat plants of 1-week post-anthesis by placing a plastic film supported by steel frames over the plots in the field at Shunyi. Wheat phenotypic data under 16 environments (E1-E16) were obtained. E1-E16 represented the environments at Shunyi in 2015 under DS + HS, DS, WW + HS and WW, at Shunyi in 2016 under DS + HS, DS, WW + HS and WW, at Changping in 2016 under WW and DS, at Shunyi in 2017 under DS + HS, DS, WW + HS and WW, at Changping in 2017 under DS and WW (Li et al., 2019a), respectively (Supplementary Table 2).

The agronomic traits including plant height (PH), spike length (SL), peduncle length (PLE), length of penultimate node (LPN), number of spikes per plant (NSP), number of spikelet per spike (NSS), number of grains per spike (NGS), and 1,000-grain weight (TGW) were measured under 16 environments. Population 2 and 3 were grown at Luoyang (36°61′N; 112°45′E) in Henan Province in 2002 and 2005, and also grown at Shunyi (40°23′N; 116°56′E) in Beijing in 2010 (Zhang et al., 2017). In all cases, each variety was planted on a 2-m, 4-row plot with 30 cm row spacing of 40 seeds in each row. Phenotypic sampling was performed for 5 plants grown in the middle of each plot at maturity.

### *TaPYL4* Gene Cloning and Construction of a Phylogenetic Tree

The reference sequence (Accession number MG273654 in the NCBI database) of *TaPYL4* was obtained according to Mega’s research (Mega et al., 2019). Genome-specific primers 2A-F1/R1, 2B-F1/R1, and 2D-F1/R1 were designed to amplify the promoter and coding region sequences of Chinese Spring wheat using Primer Premier 5.0 software (Supplementary Table 3). MEGA7 software was used to construct a neighbor joining phylogenetic tree.

### Sequence and Single Nucleotide Polymorphism Analysis of *TaPYL4s*

All genomic DNA used in this study was extracted from the wheat by CTAB method (Stewart and Via, 1993). Gene promoter and coding region sequences of *TaPYL4s* were amplified from the 32 diverse wheat accessions (Li et al., 2019b), and subsequently ligated into pEASY-Blunt vector, respectively (Trans Gen Biotech, Beijing). Positive clones were randomly selected, and the plasmid from each clone was extracted for sequencing.

According to the sequence information of *TaPYL4-2A*, *TaPYL4-2B*, and *TaPYL4-2D*, genome-specific sequencing primers CX-A-R1/R2, CX-B-R1/R2, and CX-D-F1/R1 (Supplementary Table 3) were designed by Primer 5 and using a 3730 XI DNA Analyzer (ABI) sequencing. Seqman software in DNASTAR Lasergene 7.1 package was used for sequence splice and assembly. Meg Align software was used for sequence polymorphism analysis.

### Development of Functional Molecular Markers for *TaPYL4-2A* and *TaPYL4-2B*

According to the two polymorphic sites detected, two molecular markers (TaPYL4-2A-dCAPS and TaPYL4-2B-dCAPS) were established at A genome −1635 bp (G/A) and B genome −1146 bp (G/C) upstream of the ATG translation start site, respectively. These dCAPS primers were designed with an available program dCAPS Finder 2.0. The primers were named 2A-*Sal*I-F/R and 2B-*Bam*HI-F/R, respectively (Supplementary Table 3). The first PCR product obtained with A genome-specific primers and B genome-specific primers, respectively, were served as the template for the second round of PCR. The annealing temperature and elongation time were determined by primer pairs and lengths of the expected PCR products. The PCR products were digested with the appropriate restriction enzyme at 37°C for 1.5 h and further separated on 4% agarose gel electrophoresis.

### Association Analysis

Genotype scanning was performed on the Population 1 using the molecular markers (*TaPYL4-2A*-dCAPS and *TaPYL4-2B*-dCAPS) developed. Population 1 was performed on Wheat 660 K SNP Array, which consisted of 630,517 SNPs (Li et al., 2019b). By removing nucleotide variations with missing rates ≥ 0.2 and minor allele frequency (<0.05), 395,681 SNPs were eventually used to detect the structure of population 1 by software STRUCTURE 2.3.4 (Li et al., 2019a). Using the general liner model (GLM) of TASSEL (Version 5.2.51) software and population structure (Q) as covariable, correlation analysis was conducted for phenotypic traits and genotypes of *TaPYL4-2A* and *TaPYL4-2B*. Associations were considered as significant at *P* < 0.05. Analysis of variance by SPSS (version 19.0) software was used to detect different effects of haplotype on the agronomic traits.

### Prediction of *Cis*-Acting Element in Promoter of *TaPYL4-2A* and *TaPYL4-2B*

Based on the above sequencing results, analysis of *cis*-acting element in Plant CARE^1^ was employed to predict the *cis*-acting element in the promoter region of around 2,000 bp upstream of *TaPYL4-2A* and *TaPYL4-2B* genes, respectively.

### Expression of *TaPYL4-2A* in Wheat

The expression pattern of *TaPYL4-2A* was analyzed using the wheat accession Chinese Spring. The spatiotemporal expression patterns of *TaPYL4-2A* were examined for different tissues (shoot and root at seedling stage, flag leaves, spikes, penultimate nodes, root bases, and root at heading stage). Twelve accessions of two different haplotypes were randomly selected from Population 1 (Supplementary Table 4). The gene expression levels were evaluated in shoot (St) for 20 d-old seedlings. Three biological replications were performed with each repeat at least three technical duplications.

### Yeast One-Hybrid Assays

Yeast one-hybrid assays were employed to verify the binding of TaARF4 to *TaPYL4-2A* in different haplotype promoter regions. *Not*I was used to digest the pB42AD vector to obtain *TaARF4* fragment with a restriction enzyme site. Next, the *TaARF4* was cloned into the vector with seamless clonal enzyme. Two fragments of different haplotype promoter regions of *TaPYL4-2A* (−1890 to −1440 bp) were cloned into pLacZi as the reporter gene plasmid, respectively. The plasmid of pB42AD carrier with *TaARF4* was co-transformed into EGY48 in yeast with 2 reporters by standard yeast transformation method, respectively (Lin et al., 2007). The yeast was grown on SD/-Trp/-Ura medium with x-gal. LacZ staining was used to detect the interaction between TaARF4 and promoter fragments (Wang et al., 2016). This experiment was conducted in triplicate.

### Dual-Luciferase Assays of Transformed Tobacco Leaves

Primers TaARF4-1,300-F/R (Supplementary Table 3) were used to amplify *TaARF4* full-length cDNA. The cDNA was further cloned into effector vector pCAMBIA1300 driven by CaMV35S. *TaPYL4-2A* promoter fragments of different haplotypes were amplified by primers PYL4-LUC-F1/R1 (Supplementary Table 3) and then cloned into the reporting vector pGreenII0800-LUC. The plasmid of pCAMBIA1300 vector with *TaARF4* and different reporter genes were separately transformed into GV3101 cells containing pSoup. The *Agrobactium* GV3101 containing the recombinant vector was used to transform tobacco leaves of 4 weeks by co-osmosis. The luciferase activity of firefly luciferase (LUC) and Renilla luciferase (REN) were determined at 48 h after osmosis using a Dual Glo^®^ Luciferase Assay System (E2920, Promega) with a multimode reader (TriStar^2^ S LB942). The ratio of LUC to REN was used to calculate promoter activity.

## Results

### Cloning and Molecular Characterization of *TaPYL4s* in Common Wheat

Three sub-genomic sequences of *TaPYL4* gene were isolated from a wheat accession, the Chinese Spring, namely as *TaPYL4-2A*, *TaPYL4-2B*, and *TaPYL4-2D*, respectively. The gene coding regions from A, B, and D sub-genomes are 654, 654, and 645 bp in length, respectively. Like other PYL family members, TaPYL4 proteins were highly conserved (Supplementary Figure 1A). The phylogenetic tree showed that the *TaPYL4s* were more similar to OsPYL4 (Supplementary Figure 1B).

### SNPs of *TaPYL4s* and Functional Molecular Markers Developed for *TaPYL4-2A* and *TaPYL4-2B*

**FIGURE 1:**
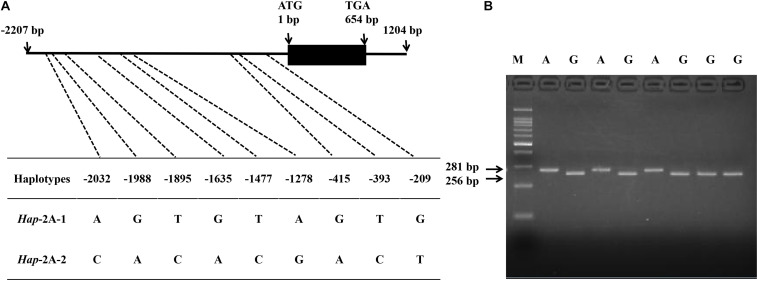
Nucleotide polymorphisms and development of a molecular marker in wheat *TaPYL4-2A*. **(A)** Nine single nucleotide polymorphisms (SNPs) were detected in the promoter region of *TaPYL4-2A*. **(B)** dCAPS marker was developed based on the –1635 bp single-nucleotide polymorphism (G/A) with restriction endonuclease *Sal*I, which cleaved the sequence only when the site was G. M, 100-bp DNA ladder.

**FIGURE 2:**
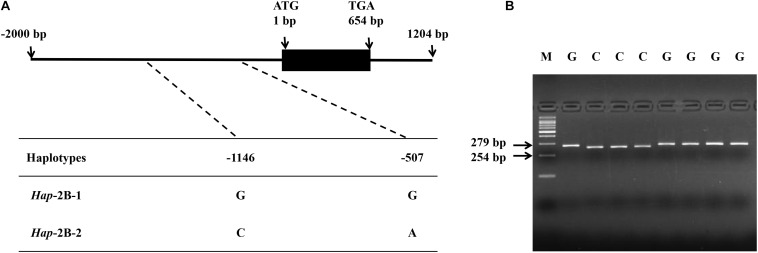
Nucleotide polymorphisms and development of a molecular marker in wheat *TaPYL4-2B*. **(A)** Two single nucleotide polymorphisms (SNPs) were detected in the promoter region of *TaPYL4-2B*. **(B)** dCAPS marker was developed based on the –1146 bp SNP (G/C) with restriction endonuclease *Bam*HI, which cleaved the sequence only when the site was C. M, 100-bp DNA ladder.

In the present study, SNPs in the coding and promoter regions of *TaPYL4-2A*, -*2B*, and -*2D* genes were detected by sequencing 32 diverse wheat accessions. No SNP was identified in the coding regions of *TaPYL4-2A* and *TaPYL4-2B.* However, nine SNPs were observed in *TaPYL4-2A* promoter region while two SNPs existed in *TaPYL4-2B* promoter region. For *TaPYL4-2D*, neither coding sequence nor promoter region was detected to have SNP. Therefore, no further investigation was on *TaPYL4-2D* in this study. According to the SNPs in *TaPYL4-2A* (Figure 1A) and *TaPYL4-2B* (Figure 2A) promoter regions, two haplotypes were identified for *TaPYL4-2A* (*Hap*-2A-1 and *Hap*-2A-2) and *TaPYL4-2B* (*Hap*-2B-1 and *Hap*-2B-2), respectively.

To accurately distinguish two haplotypes of *TaPYL4-2A*, a molecular marker (dCAPS) was developed based on the SNP (G/A) at −1635 bp of the gene, namely as *TaPYL4-2A*-dCAPS. The marker included two mismatches (AC/TT) in the downstream primer that generated the recognition site for the restriction enzyme *Sal*I (Figure 1B). In a similar way, a dCAPS marker was also developed with restriction enzyme *Bam*HI based on *TaPYL4-2B*, namely as *TaPYL4-2B*-dCAPS (Figure 2B).

### Association Analysis of *TaPYL4-2A* Haplotypes With Agronomic Traits

**FIGURE 3:**
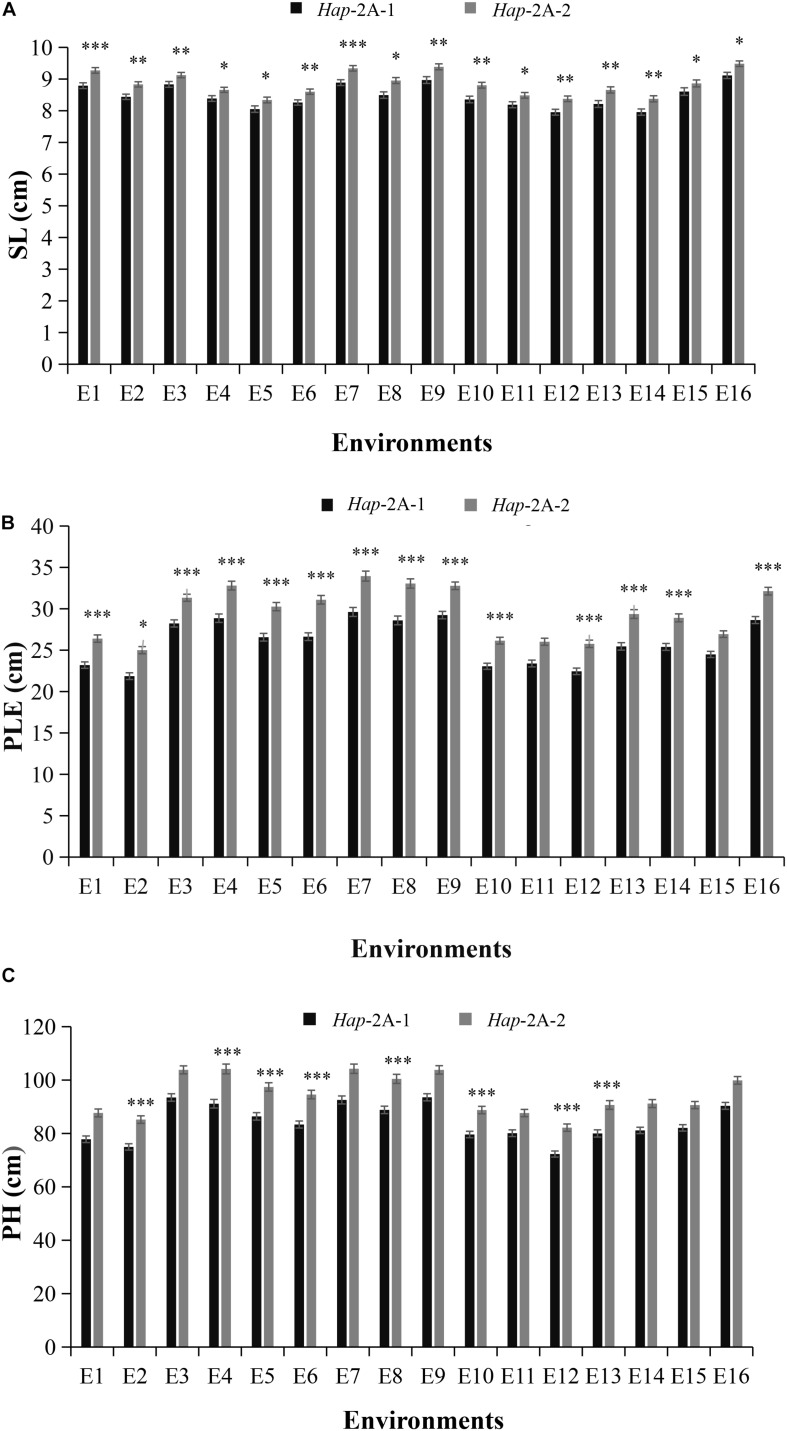
Comparisons of agronomic traits in the two wheat *TaPYL4-2A* haplotypes *Hap*-2A-1 and *Hap*-2A-2. SL: spike length **(A)**; PLE: peduncle length **(B)**; PH: plant height **(C)**. E1–E16 indicated the environments of 2015-SY-DS-HS, 2015-SY-DS, 2015-SY-WW-HS, 2015-SY-WW, 2016-SY-DS-HS, 2016-SY-DS, 2016-SY-WW-HS, 2016-SY-WW, 2016-CP-WW, 2016-CP-DS, 2017-SY-DS-HS, 2017-SY-DS, 2017-SY-WW-HS, 2017-SY-WW, 2017-CP-DS, 2017-CP-WW, respectively. SY, Shunyi; CP, Changping; DS, drought stress; HS, heat stress; WW, well-watered; ^∗^*P* < 0.05, ^∗∗^*P* < 0.01, and ^∗∗∗^*P* < 0.001, respectively. Error bars denote ± SE.

Association analysis was conducted on agronomic characters of Population 1 under 16 environments in three growth seasons. For *TaPYL4-2A*, *Hap*-2A-1 accounted for 48.9% in the Population 1 (Supplementary Figure 2). The association analysis of *TaPYL4-2A* haplotypes and agronomic traits revealed that *TaPYL4-2A* was significantly associated with SL, PLE, and PH (Table 1). The statistical analysis showed that the SL of *Hap*-2A-1 was significantly shorter than that of *Hap*-2A-2 under 16 environmental conditions (Figure 3A). Under 14 environmental conditions, the PLE of *Hap*-2A-1 was significantly shorter than that of *Hap*-2A-2 (Figure 3B). Under eight environmental conditions, the PH of *Hap*-2A-1 was significantly shorter than that of *Hap*-2A-2 (Figure 3C). In summary, the values measured for plant growth related traits of *Hap*-2A-1 were lower than that of *Hap*-2A-2.

**TABLE 1:** *TaPYL4-2A* haplotypes associated with agronomic traits across 16 environments.

**Environment**	**SL**	**PLE**	**PH**	**Environment**	**SL**	**PLE**	**PH**
	***P-*value**	***P-*value**	***P-*value**		***P-*value**	***P-*value**	***P-*value**
2015-SY-WW-HS	0.00253**	0.00967**	n.s	2015-SY-DS-HS	6.21E-05***	0.03124*	n.s
2015-SY-WW	0.00859**	0.02429*	0.03729*	2015-SY-DS	0.00150**	0.02430*	0.01396*
2016-SY-WW-HS	2.76E-04***	0.02109*	n.s	2016-SY-DS-HS	0.00442**	0.00876**	0.02804*
2016-SY-WW	4.39E-04***	0.00569**	0.03819*	2016-SY-DS	0.00214**	7.60E-04***	0.02648*
2016-CP-WW	2.39E-04***	0.01006*	n.s	2016-CP-DS	4.01E-04***	0.00855**	0.04638*
2017-SY-WW-HS	0.00196**	0.01231*	n.s	2017-SY-DS-HS	0.00847**	n.s	n.s
2017-SY-WW	0.00253**	0.01257*	0.03924*	2017-SY-DS	0.00145**	0.00926**	0.01551*
2017-CP-WW	5.70E-04***	0.01732*	n.s	2017-CP-DS	0.00806**	n.s	n.s

SY, Shunyi; CP, Changping; DS, drought stress; HS, heat stress; WW, well-watered; SL, spike length; PLE, peduncle length; PH, plant height; n.s, not significant. *P < 0.05, **P < 0.01, and ***P < 0.001.

### Association Analysis of *TaPYL4-2B* Haplotypes With Agronomic Traits

**FIGURE 4:**
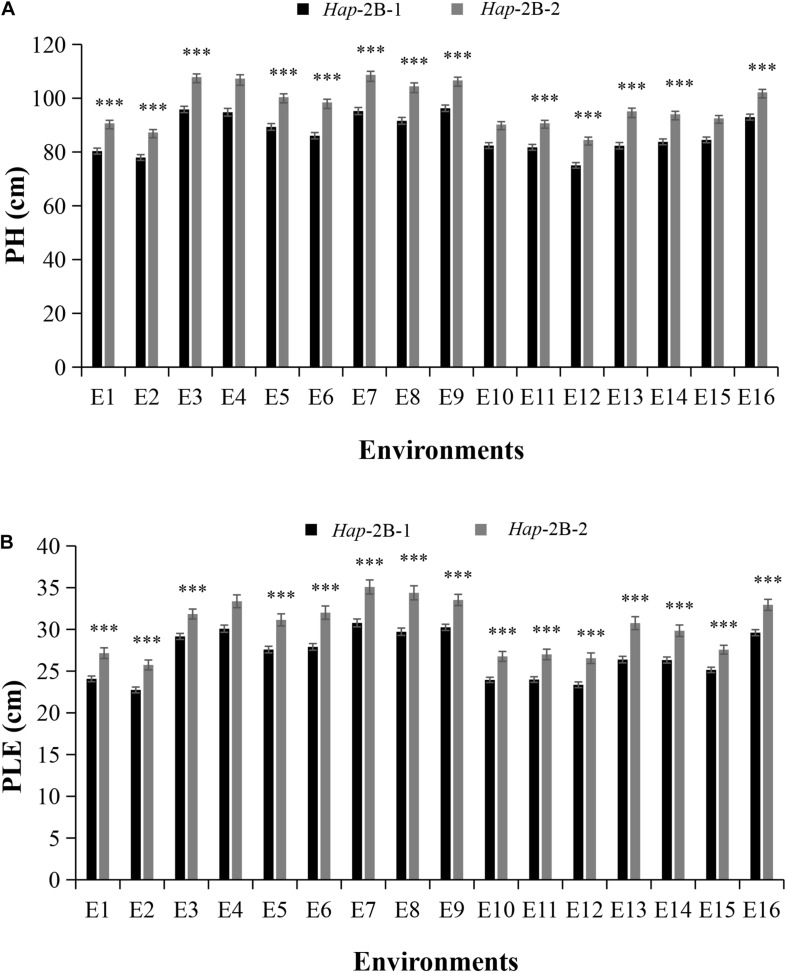
Comparisons of agronomic traits in the two wheat *TaPYL4-2B* haplotypes *Hap*-2B-1 and *Hap*-2B-2. Agronomic traits included PH **(A),** PLE **(B)**. See footnote to Figure 3 for abbreviations. ***P < 0.001. Error bars denote ± SE.

For *TaPYL4-2B*, *Hap*-2B-1, and *Hap*-2B-2 accounted for 75.5 and 24.5% in Population 1 (Supplementary Figure 2), respectively. Significant association between *TaPYL4-2B* haplotypes and PH was identified under 13 environments (Table 2). The PH of *Hap*-2B-1 was significantly shorter than that of *Hap*-2B-2 (Figure 4A). Similarly, *TaPYL4-2B* haplotypes were associated with PLE. *Hap*-2B-1 had significantly shorter PLE compared with *Hap*-2B-2 in 14 environments (Figure 4B). According to the description above, the values measured for plant growth related traits of *Hap*-2B-1 were lower than that of *Hap*-2B-2.

**TABLE 2:** *TaPYL4-2B* haplotypes associated with agronomic traits across 16 environments.

**Environment**	**PH**	**PLE**	**Environment**	**PH**	**PLE**
	***P-*value**	***P-*value**		***P-*value**	***P-*value**
2015-SY-WW-HS	0.00141**	0.01614*	2015-SY-DS-HS	0.00734**	0.00340**
2015-SY-WW	n.s	n.s	2015-SY-DS	0.03998*	0.01702*
2016-SY-WW-HS	0.00136**	0.00721**	2016-SY-DS-HS	0.01330*	0.00412**
2016-SY-WW	0.00217**	0.00134**	2016-SY-DS	5.49E-04***	0.00172**
2016-CP-WW	0.01378*	0.00758**	2016-CP-DS	n.s	0.00792**
2017-SY-WW-HS	7.38E-04***	1.54E-04***	2017-SY-DS-HS	0.03369*	0.00205**
2017-SY-WW	0.00479**	9.98E-04***	2017-SY-DS	0.01239*	0.00214**
2017-CP-WW	0.04709*	0.00571**	2017-CP-DS	n.s	0.00913**

SY, Shunyi; CP, Changping; DS, drought stress; HS, heat stress; WW, well-watered; SL, spike length; PLE, peduncle length; PH, plant height; n.s, not significant. *P < 0.05, **P < 0.01, and ***P < 0.001.

### *TaPYL4-2A* and *TaPYL4-2B* Haplotypes Have Additive Effects on Plant Growth Related Traits

**FIGURE 5:**
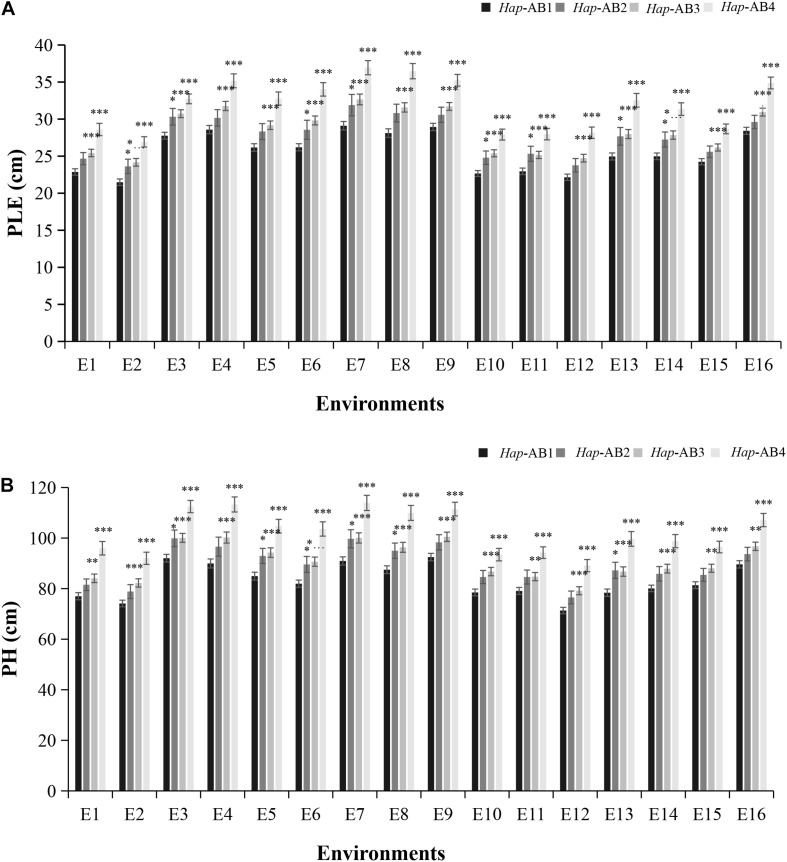
Combination of the *TaPYL4-2A* and *TaPYL4-2B* haplotypes with different effects in plant growth. Association of combinational haplotypes (*Hap*-AB1∼*Hap*-AB4) with PH **(A)** and PLE **(B)** in Population 1. ^∗^*P* < 0.05, ^∗∗^*P* < 0.01, and ^∗∗∗^*P* < 0.001, respectively. Error bars denote ± SE.

Since both *TaPYL4-2A* and *TaPYL4-2B* were associated with plant growth related traits (PH and PLE), we speculated that the *TaPYL4-2A* and *TaPYL4-2B* haplotypes may have a combinational effect on plant growth related traits. The present association studies showed that both *TaPYL4-2A* and *TaPYL4-2B* haplotypes can be divided into low PH and PLE haplotypes (*Hap*-2A-1 and *Hap*-2B-1) and high PH and PLE haplotypes (*Hap*-2A-2 and *Hap*-2B-2). Wheat accessions presenting these four combinations were divided into groups of low-/ low-, low-/ high-, high-/ low-, and high-/high-PH and PLE haplotypes, which were named as *Hap*-AB1, *Hap*-AB2, *Hap*-AB3, and *Hap*-AB4 (Supplementary Table 5), respectively. Among the four combinations, *Hap*-AB1 was associated with the lowest PLE (Figure 5A) and PH (Figure 5B) in Population 1, while *Hap*-AB4 associated with the highest PH and PLE. The plant growth related traits of *Hap*-AB2 was lower than that of *Hap*-AB3. These results demonstrated the *TaPYL4-2A* haplotypes had a larger contribution to plant growth related traits than the *TaPYL-2B* haplotypes’ effects. Furthermore, an additive effect of the *TaPYL4-2A* and *TaPYL4-2B* haplotypes was detected to work on the regulation of plant growth.

### *Cis*-Acting Element Analysis of Promoters of Two Different Haplotypes in *TaPYL4-2A* and *TaPYL4-2B*

*Cis*-acting elements in promoter region could reflect the function and regulatory mechanism of the genes. In order to further clarify the influence of polymorphic sites located in *TaPYL4-2A* and *TaPYL4-2B* promoter region, we used Plant CARE^2^ to analyze the *cis*-acting elements in promoters of two haplotypes detected for *TaPYL4-2A* and *TaPYL4-2B*, respectively. Interestingly, an ARF binding site, TGA-element (AACGAC), was detected at −1635 bp upstream of the ATG translation starting point of *Hap*-2A-1. This *cis*-acting element was absent in the promoter of *Hap*-2A-2 due to a nucleotide variation (Supplementary Figure 3). However, in the promoter regions of two haplotypes of *TaPYL4-2B*, there was no difference in *cis*-acting elements related to plant growth and development (Supplementary Figure 4).

### Expression Pattern of *TaPYL4-2A* in Various Wheat Tissues

**FIGURE 6:**
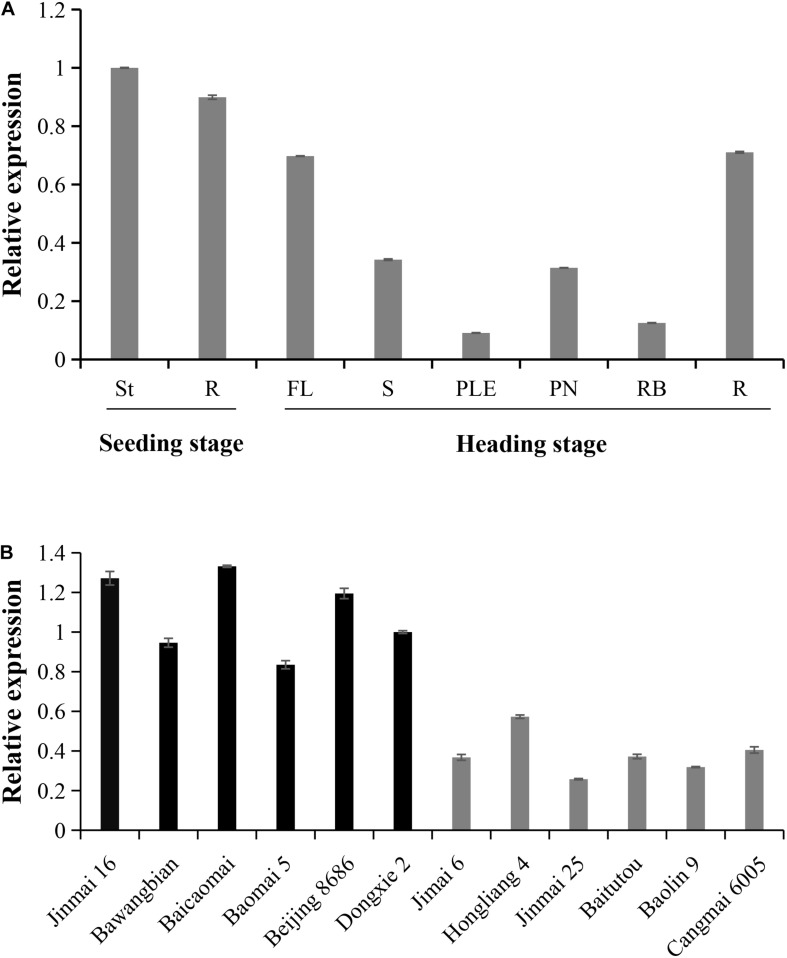
Expression patterns of the wheat *TaPYL-2A* haplotypes *Hap*-2A-1 and *Hap*-2A-2. **(A)** Tissue expression patterns of *TaPYL4-2A* were detected in the Chinese Spring by real-time PCR. St, shoot; R, root; at seedling stage. FL, flag leaves; S, spikes; PN, penultimate nodes; RB, root bases; R, root; at heading stage. The values were relative to St, which was set as 1. Error bars denote ± SE. **(B)** Expression pattern of *TaPYL-2A* in two genotype accessions at seedling stage. Jinmai 16, Bawangbian, Baicaomai, Baomai 5, Beijing 8686, and Dongxie 2 belong to *Hap*-2A-1. Jimai 6, Hongliang 4, Jinmai 25, Baitutou, Baolin 9, and Cangmai 6005 belong to *Hap*-2A-2. The values were relative to Dongxie 2, which was set as 1. Error bars denote ± SE.

Since the nucleotide variation site is located in the *TaPYL4-2A* promoter region, we further examined the impacts of this variation on the gene expression. The expression pattern of *TaPYL4-2A* gene was identified by real-time PCR at different stages of growth, showing that *TaPYL4-2A* was constitutively expressed in various tissues tested (Figure 6A).

The 12 wheat accessions representing two haplotypes of *TaPYL4-2A* were randomly selected from Population 1 and planted in the field. After 20 days of growth, shoot samples were taken for measuring the expression of *TaPYL4-2A*. Notably, the expression level of *TaPYL4-2A* in *Hap*-2A-1 wheat accessions was higher than that in *Hap*-2A-2 wheat accessions (Figure 6B).

Association analysis and expression patterns indicated that the expression level of *TaPYL4-2A* was high in *Hap*-2A-1 wheat accessions with shorter SL and PLE as well as shorter PH. On the contrary, the low expression of *Hap*-2A-2 was detected in the accessions with longer SL and PLE as well as taller PH. Therefore, *TaPYL4-2A* gene expression might be negatively correlated with plant growth-related traits.

### *TaARF4* Regulates *TaPYL4-2A* Expression by Binding to the Promoter Region

**FIGURE 7:**
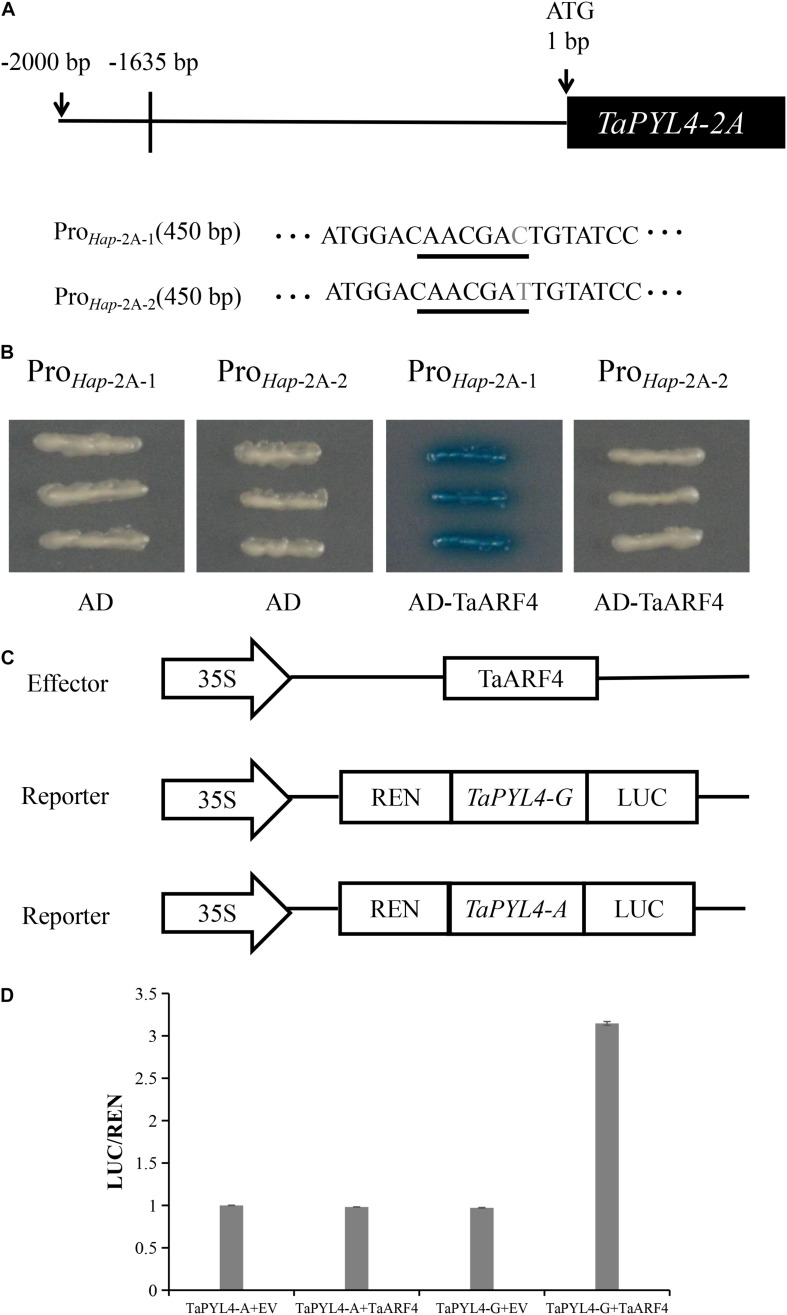
TaARF4 binds to the wheat *TaPYL4-2A* of *Hap*-2A-1 promoter region. **(A)** Schematic diagram of *Hap*-2A-1 of *TaPYL4-2A*. Underlined letters indicated the TGA-element. Letter in gray was the single-nucleotide polymorphism site (G/A). **(B)** Yeast one-hybrid assays of TaARF4 and *TaPYL4-2A* two haplotype promoters. **(C)** Dual-luciferase assay of transformed tobacco leaves to detect the interaction between TaARF4 and the *TaPYL4-2A* promoter. Schematic diagrams of the effector and reporter constructs are performed. *TaARF4* was cloned into the effector construct pCAMBIA1300 and the *TaPYL4-2A* two haplotype promoters were inserted into the reporter vector pGreen II 0800-LUC. Agrobacterium combine with vector pSoup containing reporter and effector or with empty carrier (EV) after transformation, infiltrate into leaves together. **(D)** Promoter activities were shown as the ratio of LUC to REN. Data are means (± SE) of three biological replicates.

Previous reports showed that ARF7 and ARF19 could be bound to the TGA-element in *Arabidopsis* (Huang et al., 2018). *TaARF4* was related to plant height in wheat (Wang et al., 2019b). Therefore, we speculated that *TaARF4* may regulate the expression of *TaPYL4-2A.* Yeast one-hybrid assays were carried out to determine whether TaARF4 binds to the promoter region of *TaPYL4-2A*. The *TaARF4* DNA binding region was cloned into pB42AD vector. The promoter regions of the haplotype *TaPYL4-2A* with and without the TGA-element were cloned into the pLacZi, separately (Figure 7A). In order to identify the interaction between promoters and TaARF4, the plasmid containing each of these 2 promoter regions was transformed into yeast EGY48 with *TaARF4* plasmid, respectively. Positive yeast EGY48 cells were cultured on the selective medium of x-gal without Ura and Trp. In the presence of TGA-element in *Hap*-2A-1 of *TaPYL4-2A*, TaARF4 were successfully bound to the promoter to activate the expression of LacZ reporter gene. However, in the absence of TGA-element in *Hap*-2A-2 of *TaPYL4-2A*, TaARF4 could not bind the promoter to activate the LacZ reporter gene (Figure 7B). The results indicate that due to the difference of A/G at −1635 bp upstream of ATG translation starting point, TaARF4 could bind to the promoter region of *Hap*-2A-1, but not to the promoter region of *Hap*-2A-2.

We also performed a LUC experiment for further verification. Dual luciferase assays illustrated that LUC activity was higher than that of the negative control in the presence of TaARF4 effector and *TaPYL4-2A* promoter containing TGA-element (Figures 7C,D). On the contrary, LUC activity was similar to that of the negative control in the presence of TaARF4 effector and *TaPYL4-2A* promoter without the TGA-element. These results again indicated that TaARF4 functions as a transcription activator for *Hap-*2A-1, but not for *Hap-*2A-2.

### Geographical and Temporal Distribution of *TaPYL4-2A* and *TaPYL4-2B* Haplotypes Among the Wheat Varieties Cultivated in China

**FIGURE 8:**
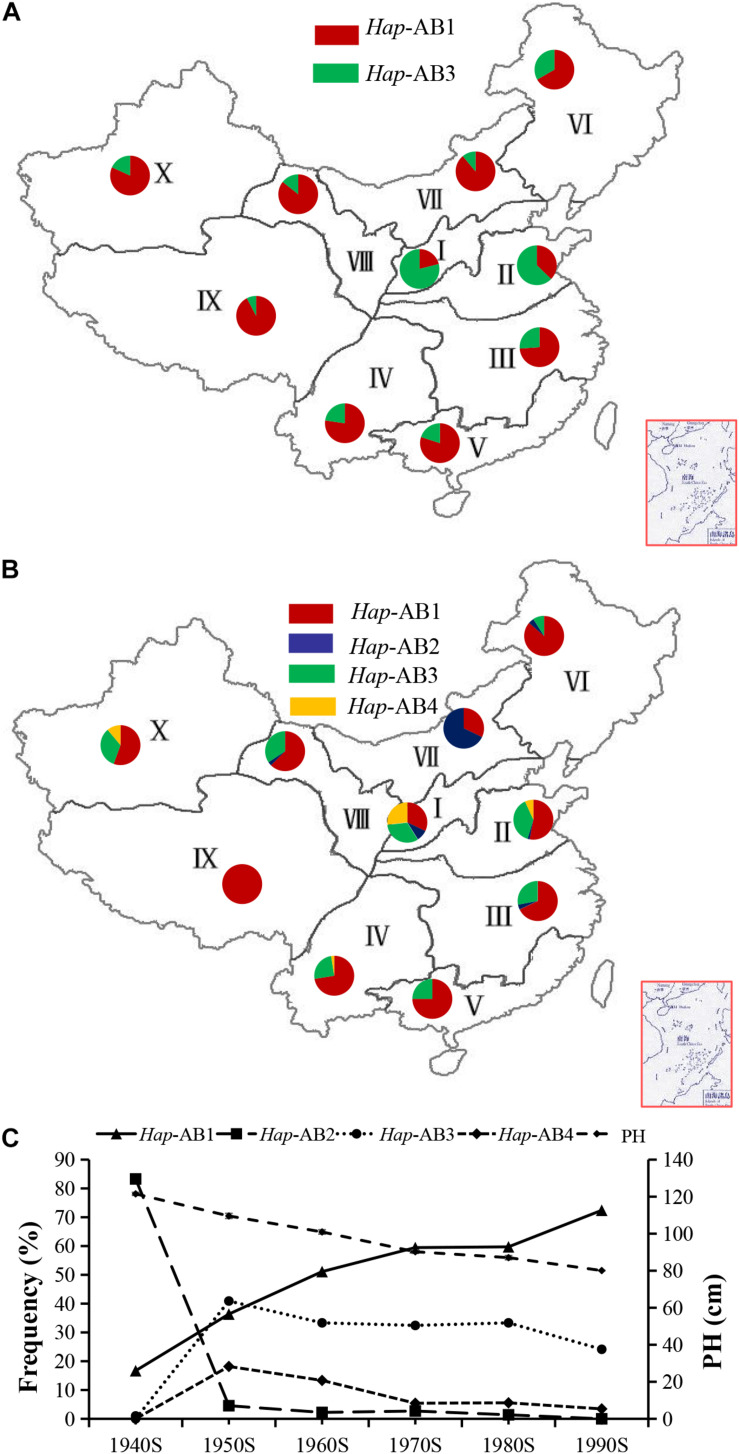
Haplotype distribution and frequency change of *TaPYL4-2A* and *TaPYL4-2B* in 10 wheat-producing regions across China. **(A)** Distributions of *TaPYL4-2A* and *TaPYL4-2B* haplotypes (*Hap*-AB1∼*Hap*-AB4) in 157 Chinese landraces. **(B)** Distributions of *TaPYL4-2A* and *TaPYL4-2B* haplotypes (*Hap*-AB1∼*Hap*-AB4) in 348 modern cultivars. I, Northern winter wheat region; II, Yellow and Huai River valley winter wheat region; III, Low and middle Yangtze River valley winter wheat region; IV, Southwestern winter wheat region; V, Southern winter wheat region; VI, Northeastern spring wheat region; VII, Northern spring wheat region; VIII, Northwestern spring wheat region; IX, Qinghai-Tibet spring-winter wheat region; X, Xinjiang winter-spring wheat region. **(C)** Haplotype frequency and PH (cm) change of *Hap*-AB1∼*Hap*-AB4 in 348 modern cultivars of Chinese major wheat zones. The data for PH (cm) are from Hao et al. (2011a).

According to the regional climate characteristics, topography, cultivation characteristics, sowing and maturity stage, Chinese wheat planting regions were divided into 10 major ecological areas (Zhang et al., 2002). From Chinese landraces to modern cultivars, the proportion of *Hap*-AB1 was increased significantly in regions I, II, VI, and IX, but not significantly changed in wheat regions III, IV, and V (Figures 8A,B). The mean PH in modern wheat varieties released in China from the 1940s to the 1990s has been reduced from 121.50 cm to 80.08 cm, and this change was associated with the increasing of proportion of haplotype *Hap*-AB1 from 16.70 to 72.41% (Figure 8C). To sum up, *Hap*-AB1 was selected by wheat breeders in the past decades, showing a considerable utilization potential in wheat genetic improvement.

## Discussion

Since the Green Revolution, breeders have been trying to develop the ideal plant architecture to increase wheat yield. As a consequence, it is vitally significant to reveal the molecular mechanism of the plant development. In the present study, we found that *TaPYL4-2A* and *TaPYL4-2B* contributed to the growth-related traits such as spike length (SL), peduncle length (PLE) and plant height (PH) in wheat. No polymorphism was detected for *TaPYL4-2D* among the wheat accessions tested. It is unclear whether *TaPYL4-2D* affects plant height, which needs further study. Our data demonstrated that TaARF4 could activate expression of *Hap*-2A-1 of *TaPYL4-2A* by binding to the *cis*-acting element in the promoter. Therefore, *TaPYL4* might play an important role in controlling the growth and development of plants.

ABA receptors are important signaling molecules in plant ABA signaling pathways. They could activate transcription factors in downstream of signaling pathways and regulate the expression of the related genes, thus participating in plant growth and stress responses (Tian et al., 2015). Previously, *Arabidopsis* plants overexpressing *PYL9/RCAR1*, *PYL5/RCAR8*, or *PYL8/RCAR3* were reported to have a significant increase in drought tolerance and more sensitive to ABA during seed germination, vegetative growth, stomatal movement and other physiological processes (Fujii et al., 2009; Santiago et al., 2009). In rice, *OsPYL4* and *OsPYL/RCAR5* were identified to play important roles in plant development (Kim et al., 2014; Miao et al., 2018). In wheat, *TaPYL4*-overexpressed lines showed significant increase in grain yield compared to the control under the condition of insufficient water (Mega et al., 2019). More importantly, our study indicated that *TaPYL4* functions in the morphogenesis of plant growth-related traits, suggesting that *TaPYL4* can balance wheat stress resistance and plant growth.

ABA is a versatile hormone. For example, ABA and auxin could co-regulate root growth to adapt to the changing environment (De Smet et al., 2003; Overvoorde et al., 2010; Zhao et al., 2014). Auxin enhanced ABA signaling through ARF10 and ARF16-mediated activation of *ABI3* expression in regulating lateral root formation and seed dormancy (Brady et al., 2003; Liu et al., 2013). AtPYL8 was revealed to regulate lateral root growth by enhancing transcription of auxin responsive genes (Zhao et al., 2014). Recently, ARF7 and ARF19 transcription factors were examined to bind to TGA-element to regulate the expression of downstream genes (Huang et al., 2018). TaARF4 were linked to plant height (Wang et al., 2019b). Thus, it could be inferred that auxin response factors were related to ABA receptors. Our data demonstrated that TaARF4 could bind to the promoter region of *Hap*-2A-1 of *TaPYL4-2A* to activate the expression of *TaPYL4-2A* (Figure 7). In wheat materials having *Hap*-2A-1, *TaPYL4-2A* expression was high, but plant growth-related traits were low. On the contrary, *TaPYL4-2A* expression in *Hap*-2A-2 was low whereas plant growth-related traits were high (Supplementary Figure 5). These studies revealed that TaARF4 regulates *TaPYL4-2A* gene expression to affect plant growth-related traits. However, whether TaARF4 and TaPYL4-2A affect the content of ABA and auxin is still unclear. Further study needs to focus on this regard.

Association analysis showed that *TaPYL4-2B* gene was also closely related to PLE and PH. Additive effects were detected for *TaPYL4-2A* and *TaPYL4-2B.* However, no difference in *cis*-acting elements of two *TaPYL4-2B* haplotype’s promoters was found to be related to plant growth and development. More studies are needed to clarify the molecular mechanism underlying plant growth and development of *TaPYL4-2B.* At present, no polymorphism was detected for *TaPYL4-2D*, and this gene function has not been elaborated. Furthermore, *TaPYL4-2D* was structurally conservative, implying that its function may be important to other traits.

Marker-assisted selection is recognized as an efficient method to accelerate wheat breeding process (Liu et al., 2012). Here, we analyzed polymorphisms in *TaPYL4-2A* and *TaPYL4-2B*, and consequently developed the functional molecular markers to identify different haplotypes associated with plant growth-related traits. Therefore, these markers could be used in marker-assisted selection to breed wheat varieties with more suitable plant architecture despite the frequency of the favorable *Hap*-AB1 allele was increased from 16.70 to 72.41% in modern wheat varieties.

In conclusion, the coding sequence of *TaPYL4* gene (*TaPYL4-2A*, *TaPYL4-2B*, and *TaPYL4-2D*) is highly conserved. Nucleotide variations were identified in *TaPYL4-2A* and *TaPYL4-2B*’s promoter regions. Based on the SNP at position−1635 (G/A) of *TaPYL4-2A* and −1146 (G/C) of *TaPYL4-2B*, two haplotypes were detected, respectively. TaARF4 could activate the expression of *TaPYL4-2A* by binding to the promoter region of *Hap*-2A-1 of *TaPYL4-2A*. Remarkably, the combinational haplotype *Hap*-AB1 was positively associated with low PH. This *Hap*-AB1 haplotype was broadly selected in wheat breeding during the last decades. The functional marker developed for *Hap*-AB1 can be used in wheat genetic improvement to develop the ideal plant architecture with high grain yield.

## Data Availability Statement

The authors acknowledge that the data presented in this study must be deposited and made publicly available in an acceptable repository, prior to publication. Frontiers cannot accept a manuscript that does not adhere to our open data policies.

## Author Contributions

RJ, JW, and RL conceived the idea and revised the manuscript. YX, JW, XM, CL, XC, and XY performed the experiments. YX, JW, LL, XM, CH, and RJ analyzed the data. YX wrote the manuscript. All authors contributed to the article and approved the submitted version.

## Conflict of Interest

The authors declare that the research was conducted in the absence of any commercial or financial relationships that could be construed as a potential conflict of interest.

## Publisher’s Note

All claims expressed in this article are solely those of the authors and do not necessarily represent those of their affiliated organizations, or those of the publisher, the editors and the reviewers. Any product that may be evaluated in this article, or claim that may be made by its manufacturer, is not guaranteed or endorsed by the publisher.

## Footnotes

1
http://bioinformatics.pAet.ugent.be/webtools/plantcare/html

2
http://bioinformatics.psb.ugent.be/webtools/plantcare/html
