# Supplementary material for: Association Analysis Revealed That TaPYL4 Genes Are Linked to Plant Growth Related Traits in Multiple Environment
Source: Front Plant Sci. 2021 Aug 11;12:641087. doi: 10.3389/fpls.2021.641087 (PMC8387097; doi:10.3389/fpls.2021.641087)
Supplement: Supplementary file 1 [file Data_Sheet_1.docx]

Supplementary Material

# Supplementary Figures and Tables

## Supplementary Figures

**A**


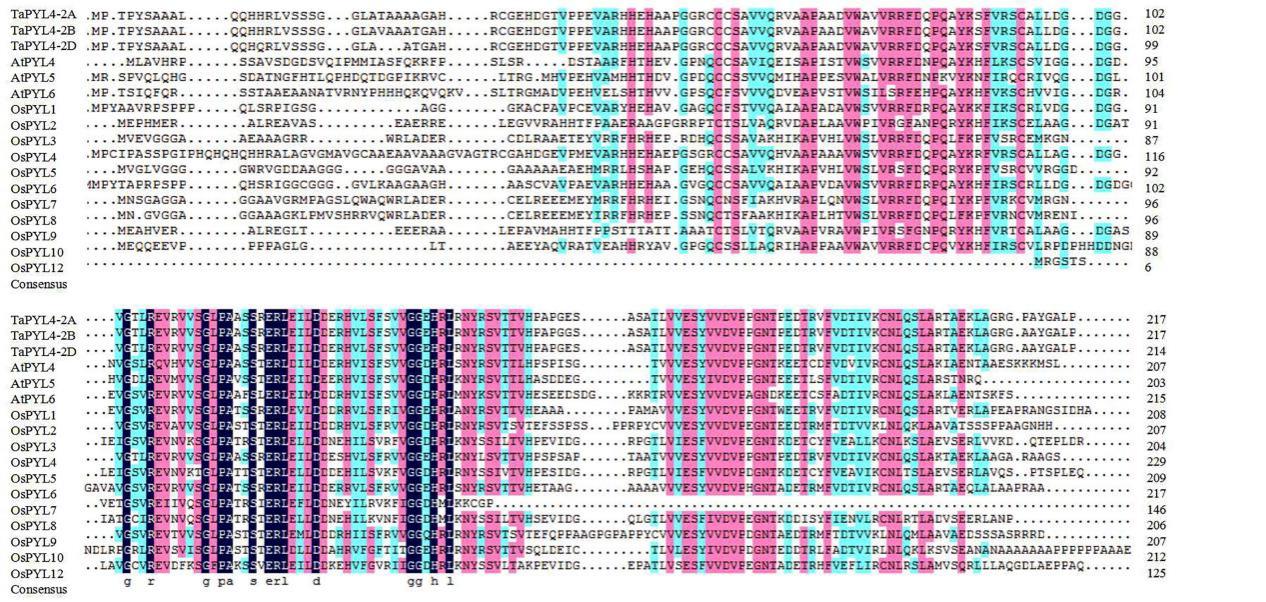


**B**


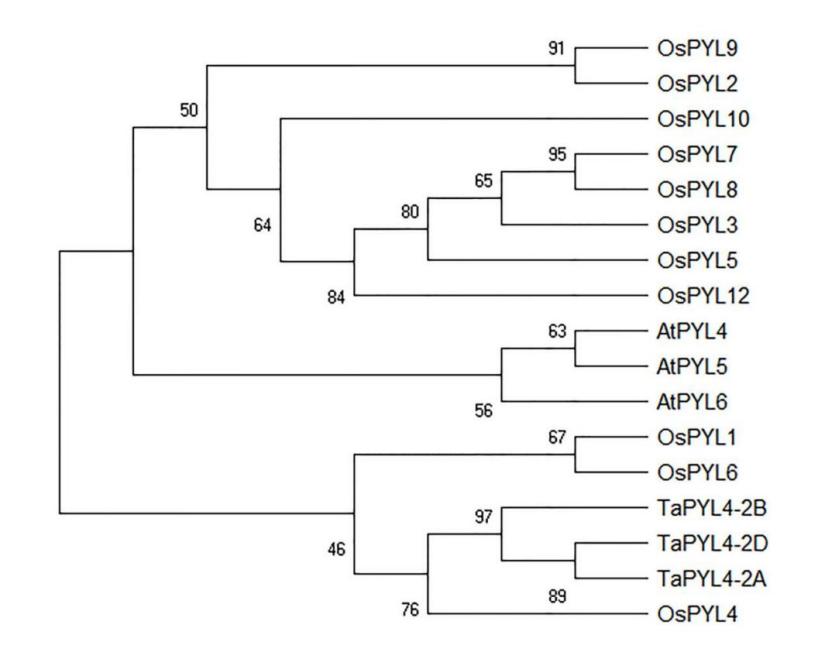


**Supplementary Figure 1** | Structural analysis and phylogenic tree of TaPYL4 protein and homologous PYLs proteins. (**A**) Amino acid sequences alignment of TaPYL4 and homologous proteins. Amino acids shaded by color are conserved, black shaded amino acids indicate the highest similarity, red less, and blue least. (**B**) Phylogenetic tree of TaPYL4s and ABA receptors in *Arabidopsis* and rice. At, *Arabidopsis thaliana*; Os, *Oryza sativa*; Ta, *Triticum aestivum*.

**Supplementary Figure 2** | Frequencies of two haplotypes of *TaPYL4-2A* and *TaPYL4-2B* in Population 1.


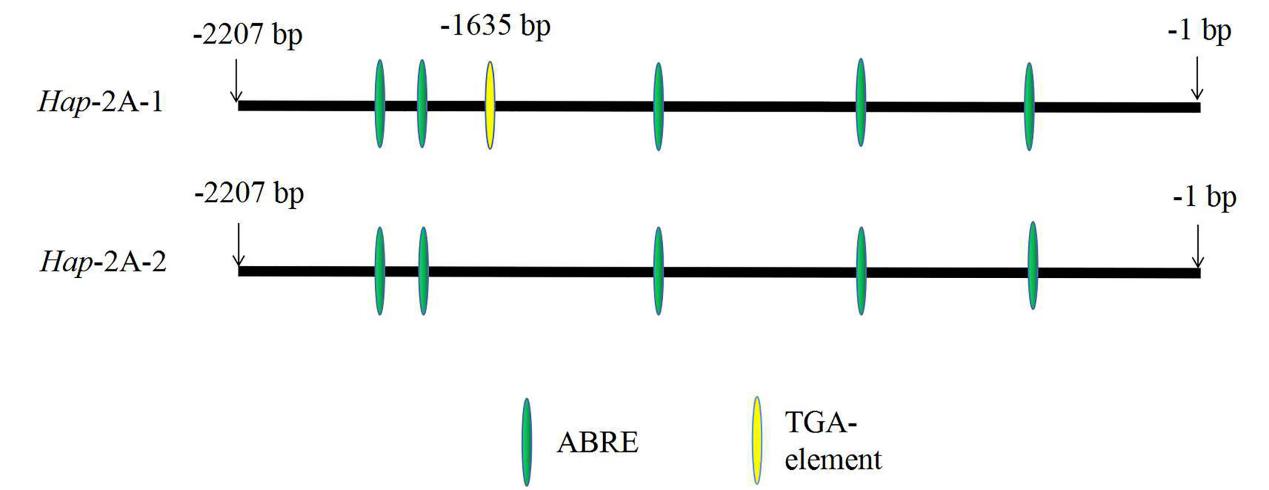


**Supplementary Figure 3** | Distributions of cis-acting elements in the upstream promoters of *TaPYL4-2A*.


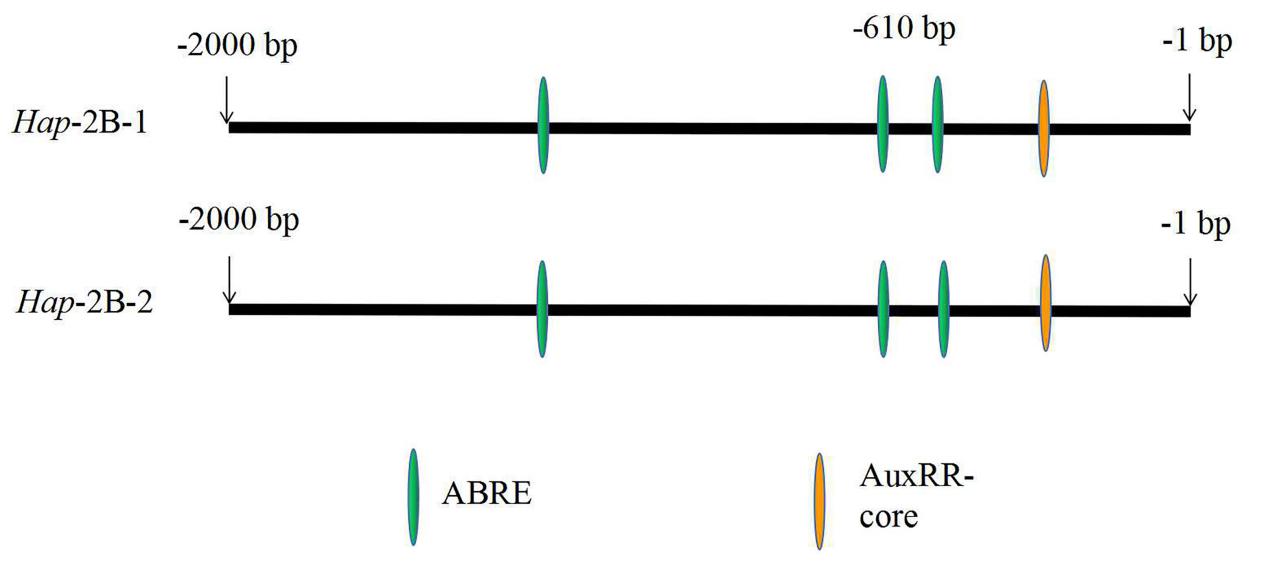


**Supplementary Figure 4** | Distributions of cis-acting elements in the upstream promoters of *TaPYL4-2B*.


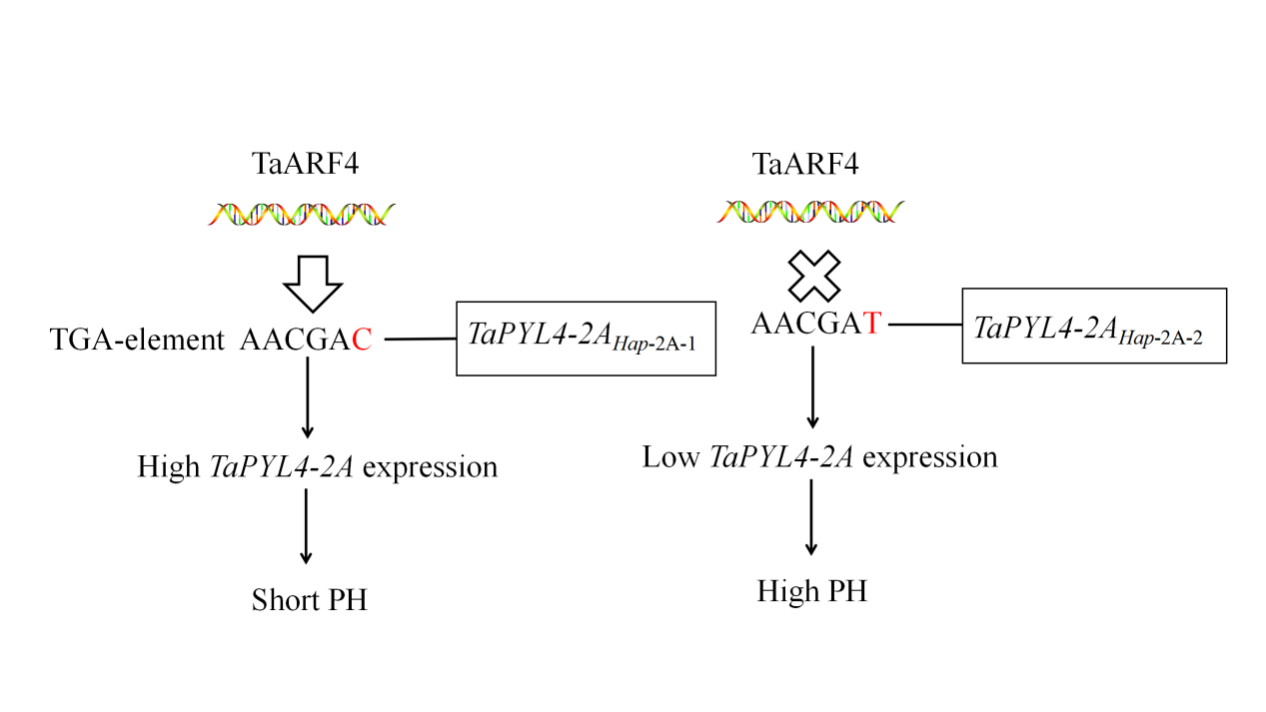


**Supplementary Figure 5** | A working model of two haplotypes of *TaPYL4-2A* and *TaARF4* relationships. PH: plant height.

## Supplementary Tables

**Supplementary Table 1 |** The information of thirty-two wheat accessions

| **Number** | **Accession** | **Origin** | **Number** | **Accession** | **Origin** |
| --- | --- | --- | --- | --- | --- |
| 1 | PANDAS | Italy | 17 | Linkang 5108 | Shanxi |
| 2 | An85 Zhong124-1 | Beijing | 18 | Baiqimai | Gansu |
| 3 | Yanzhan 1 | Henan | 19 | Changle 5 | Shandong |
| 4 | Bawangbian | Hebei | 20 | Hongheshang | Shanxi |
| 5 | Beijing 10 | Beijing | 21 | Beijing 8686 | Beijing |
| 6 | Beijing 14 | Beijing | 22 | 04-044 | Beijing |
| 7 | Cangzhouxiaomai | Hebei | 23 | 04-030 | Beijing |
| 8 | Changwu 131 | Shaanxi | 24 | Chun 22 9th-25 | CIMMITY |
| 9 | Chang 6878 | Shanxi | 25 | Ziganbaimangxian | Henan |
| 10 | Dali 1 | Shaanxi | 26 | Jingpin 10 | Beijing |
| 11 | Dan R8093 | Beijing | 27 | Chun 04 9th-5-1 | CIMMITY |
| 12 | Fengkang 13 | Beijing | 28 | Chun 45 9th-50-1 | CIMMITY |
| 13 | Jimai 41 | Hebei | 29 | Neixiang 188 | Henan |
| 14 | Jimai 6 | Hebei | 30 | Jing 411 | Beijing |
| 15 | Jin 2148-7 | Fujian | 31 | Chinese Spring | Sichuan |
| 16 | Jinghe 8922 | Beijing | 32 | Baicaomai | Henan |

**Supplementary Table 2 |** The information of 16 environments

| **Number** | **Environment** | **Number** | **Environment** |
| --- | --- | --- | --- |
| E1 | 2015-SY-DS-HS | E9 | 2016-CP-WW |
| E2 | 2015-SY-DS | E10 | 2016-CP-DS |
| E3 | 2015-SY-WW-HS | E11 | 2017-SY-DS-HS |
| E4 | 2015-SY-WW | E12 | 2017-SY-DS |
| E5 | 2016-SY-DS-HS | E13 | 2017-SY-WW-HS |
| E6 | 2016-SY-DS | E14 | 2017-SY-WW |
| E7 | 2016-SY-WW-HS | E15 | 2017-CP-DS |
| E8 | 2016-SY-WW | E16 | 2017-CP-WW |

SY: Shunyi; CP: Changping; DS: drought stress; HS: heat stress; WW: well-watered.

**Supplementary Table 3 |** Primers used for this study

| **Primer set** | **Nucleotide sequence (5' to 3' )** | **Experimental purpose** |
| --- | --- | --- |
| 2A-F1 | TGCTCGGAGATGCAGTTTGG | A genome-specific primers |
| 2A-R1 | GGAGATAATCTTGATTCAGTCCGG | A genome-specific primers |
| 2B-F1 | GGCATATTTCCTTCACTAGACACC | B genome-specific primers |
| 2B-R1 | AGGCTGGGCTTGAGTTGA | B genome-specific primers |
| 2D-F1 | GAAGGGAATCGCAATTTTTAGC | D genome-specific primers |
| 2D-R1 | CCTCGTTGAACATCTTGTAAATTTG | D genome-specific primers |
| M13F | TGTAAAACGACGGCCAGT | Sequencing primers for A/B/D genome |
| M13R | CAGGAAACAGCTATGACC | Sequencing primers for A/B/D genome |
| CX-A-R1 | AATGCATCTCTGAGCGGAGA | Sequencing primers for A genome |
| CX-A-R2 | CCTTAAAAAAAACACATTTTCCTG | Sequencing primers for A genome |
| CX-B-R1 | CAAATACGACAAGGAGCACCA | Sequencing primers for B genome |
| CX-B-R2 | ACCTGTGTGCCCCCGTCG | Sequencing primers for B genome |
| CX-D-F1 | AGGAAATGAGGACCAGCCACT | Sequencing primers for D genome |
| CX-D-R1 | GGTAACTTCCTTGTTGAAACACCTC | Sequencing primers for D genome |
| 2A-Sal I -F | CATACACGTTCGCCGAATTTC | Marker developed forSNP-1635(G/A) |
| 2A-Sal I -R | CCCCCTTGTCTAATATGGACGTCGA |  |
| 2B-BamH I-F | TCTCCGCCGCCACGAGCGAGGATC | Marker developed for SNP-1146(G/C) |
| 2B-BamH I-R | AGGACAGCGACACCACGAAC |  |
| PYL4-2A-qF1 | CACCATCGTCAAGTGCAACCT | Real-time PCR |
| PYL4-2A-qR1 | GAGCCGGGCTCGAGTTGA | Real-time PCR |
| Tubulin-qF | GAGGCCTCGTGTGGTCGCTTTGT | Real-time PCR |
| Tubulin-qR | GCCCAGTTGTTACCCGCACCAGA | Real-time PCR |
| ARF4-pB42AD-F | TGCCTCTCCCGAATTCATGGCGCCGCCGCAGGCC | pB42AD vector construction |
| ARF4-pB42AD-R | CGAGTCGGCCGAATTCTCAAATCGGCTTAACGCAACCTTC | pB42AD vector construction |
| PYL4-2A-LacZi-F | ATCTGTCGACCTCGAGCATACACGTTCGCCGAATTTC | pLacZi construction |
| PYL4-2A-LacZi-R | GAGCACATGCCTCGAGAATGCATCTCTGAGCGGAGA | pLacZi construction |
| PYL4-LUC-F1 | CGGTATCGATAAGCTTTGGGTAGAAAGAGATGAGACAGTGA | Reporter Vector |
| PYL4-LUC-R1 | TTGGCGTCTTCCATGGCCCCGTCTCCTCTCTCCCTTAA | Reporter Vector |
| TaARF4-1300-F | CTAGTCTAGAATGGCGCCGCCGCAGGCC | Effector Vector |
| TaARF4-1300-R | TGACACTAGTGAATCGGCTTAACGCAACCTTCCTC | Effector Vector |

**Supplementary Table 4 |** The information of 12 wheat accessions in Population 1 for real-time PCR analysis

| **Number** | **Accession** | **allele** | **Number** | **Accession** | \| **allele** \| \| --- \| |
| --- | --- | --- | --- | --- | --- | --- |
| 106 | Jinmai 16 | G | 101 | Jimai 6 | A |
| 110 | Bawangbian | G | 105 | Hongliang 4 | A |
| 111 | Baicaomai | G | 107 | Jinmai 25 | A |
| 115 | Baomai 5 | G | 113 | Baitutou | A |
| 117 | Beijing 8686 | G | 114 | Baolin 9 | A |
| 145 | Dongxie 2 | G | 122 | Cangmai 6005 | A |

**Supplementary Table 5** | Combinations of *TaPYL4-2A* and *TaPYL4-2B* high-PH (plant height) and PLE (peduncle length), short-PH and PLE haplotypes (*Hap*-AB1~*Hap*-AB4). The number of *Hap*-AB1~*Hap*-AB4 is 129, 29, 115, and 50, respectively, in Population 1.

| Haplotypes | *Hap*-2A-1  Short PH, PLE | *Hap*-2A-2  High PH, PLE |
| --- | --- | --- |
| *Hap*-2B-1  Short PH, PLE | *Hap*-AB1 (n=129) | *Hap*-AB3 (n=115) |
| *Hap*-2B-2  High PH, PLE | *Hap*-AB2 (n=29) | *Hap*-AB4 (n=50) |

PH: plant height; PLE: peduncle length.
